# Refractory Pyoderma Gangrenosum: Stabilized on Colchicine and Tumor Necrosis Factor (TNF) Inhibitor

**DOI:** 10.7759/cureus.30419

**Published:** 2022-10-18

**Authors:** Salha Hakami, Yousef Alharthi, Abeer Mohammed M Alanazi

**Affiliations:** 1 Department of Dermatology, King Salman Hospital, Riyadh, SAU; 2 Department of Dermatology, King Salman Armed Forces Hospital, Tabuk, SAU; 3 Department of Dermatology, University of Tabuk, Tabuk, SAU

**Keywords:** tumor necrosis factor alpha (tnf-α), mycophenolate mofetil, colchicine, refractory, pyoderma gangrenosum

## Abstract

Pyoderma gangrenosum (PG) is a rare ulcerative, painful inflammatory skin condition that is categorized among neutrophilic dermatoses. We report an otherwise healthy female who initially presented to a dermatology clinic with erythematous nodules and pustules scattered over her legs and thigh, which progressed later to multiple painful ulcers. Upon further investigation, it was diagnosed as idiopathic PG. Prednisone was an initial mainstay of treatment. While tapering, mycophenolate mofetil was started as adjunctive therapy but failed to maintain remission. A tumor necrosis factor inhibitor was initiated alongside colchicine with a significant clinical response.

## Introduction

Pyoderma gangrenosum (PG) is an ulcerative inflammatory cutaneous disease frequently presenting as an inflammatory papule or pustule that evolves into a painful ulcer with a violaceous, eroded margin and a purulent floor. Additionally, PG may manifest with bullous, vegetative, peristomal, or extracutaneous lesions [1]. PG is an uncommon condition with an incidence between three and 10 per million individuals yearly [2]. The etiology, pathophysiology, and optimal treatment remain unclear. Therefore, variable laboratory and histopathologic data necessitate clinicopathologic correlation for diagnosis [3].

As PG is considered an inflammatory process, systemic corticosteroid therapy is the first-line therapy. Unfortunately, there is a 40% risk of relapse after complete remission [2]. Consequently, tapering corticosteroids with a steroid-sparing agent have been shown to prevent relapse [4]. Colchicine has been shown to be effective in treating neutrophilic dermatoses, such as Sweet syndrome and Behcet's disease, making it a valuable option for controlling PG [2].

After achieving remission on systemic corticosteroids but failing to respond to mycophenolate mofetil, we report a patient who was successfully stabilized on a combination of adalimumab and colchicine. To our knowledge, this is the first case in the published literature to report disease control on this treatment protocol.

## Case presentation

**Figure 1:**
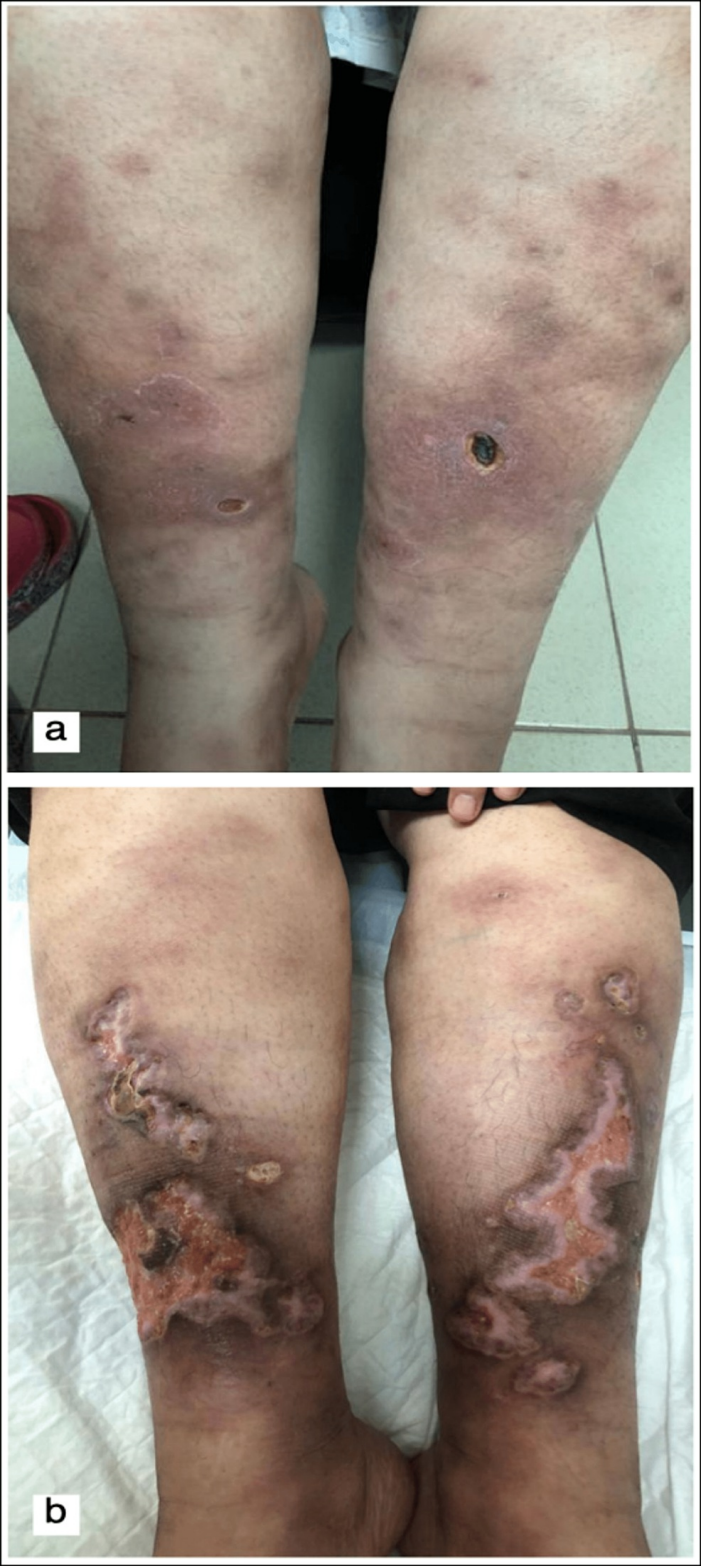
The clinical presentation of pyoderma gangrenosum (a) Erythematous nodules with small ulcers bilaterally. (b) Ulcers have progressed over the weeks.

A 42-year-old, previously healthy female presented to the dermatology clinic with erythematous nodules and pustules scattered over her legs and thighs. We presumed it was furunculosis, for which she was given antibiotics. One week later, it progressed to painful ulcers. Physical examination revealed multiple tender ulceration involving the legs bilaterally. She denied any skin trauma. The ulcerations were deep into fat with violaceous undermined borders, edematous and erythematous surrounding skin, and fibrinoid and purulence exudate at the bases, as seen in Figures 1A, 1B. Histopathologic examination revealed neutrophilic dermatosis with ulceration. Systemic evaluation of the patient did not reveal any systemic symptoms, including gastrointestinal symptoms and arthralgias, and did not suffer from any of the conditions noted to be associated with PG.

**Figure 2:**
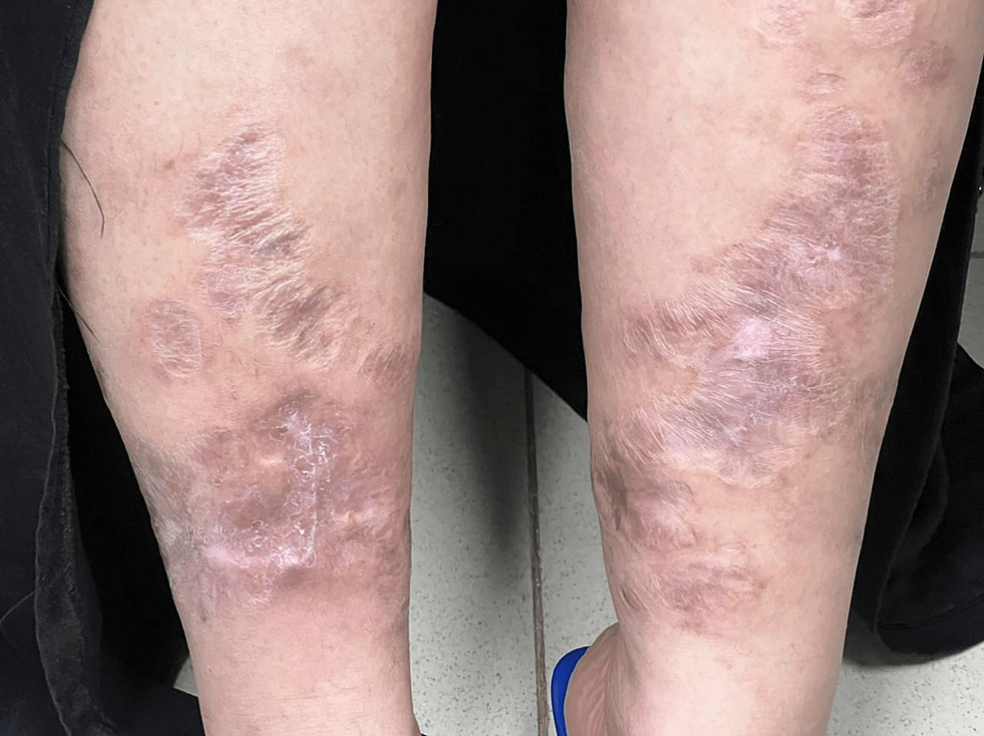
Healed pyoderma gangrenosum

Oral prednisone 60 mg daily was started for three weeks. After initial improvement, mycophenolate mofetil 3 g per day was added. Prednisone tapering was attempted twice, resulting in moderate to severe flaring of her disease. Therefore, mycophenolate mofetil was discontinued because of suboptimal response. After a verbal agreement, the patient began receiving adalimumab at 40 mg every two weeks with significant improvement. Prednisone was tapered over six weeks and then discontinued. Milder relapse was noticed as small painful nodules, intralesional triamcinolone 10mg/mL was received, and adalimumab was adjusted to 40mg per week with significant improvement. However, after improvement for two months, another mild relapse was noticed. Colchicine 2.4 mg/day, divided into two doses, was initiated. After three weeks, clear improvement and sustained remission were observed. The combination of adalimumab and colchicine proved effective. At her last follow-up after four months of therapy, lesions had improved and healed with a cribriform scar as seen in Figure 2.

## Discussion

PG was first described in 1908 as a “geometric phagedena” and was later redefined as PG in 1930. PG is a misnomer; it is neither an infectious nor gangrenous condition [5]. The etiology of PG remains uncertain in the literature; however, neutrophilic dysfunction, genetic variation, and dysregulation of the immune system are postulated as contributing factors to the pathogenesis of PG. Hence, it can be associated with systemic diseases like inflammatory bowel disease, arthritis, and hematological disorders, sometimes leading to choosing one medication over the other [6,7]. Treatment of PG remains challenging, and, to date, no guidelines have been established [2,8].

PG is considered an autoimmune disease; corticosteroids are the initial therapy to induce remission, but they carry a risk of adverse effects in the long term. Therefore, Mycophenolate mofetil (MMF) was a favorable steroid-sparing agent in refractory PG, as reported in the literature, with 84.6% of patients responding to the treatment [9]. Another literature review reported the results of multiple case series totaling 63 patients treated with MMF. The result shows that 93% of patients improved on MMF, with five patients achieving complete remission [10]. Surprisingly, our patient had several moderate-to-severe relapses when prednisone was tapered.

TNF-α inhibitors showed a promising result in treating PG. A review of case reports and series conducted in 2019 demonstrates that adalimumab has a success rate of 90%. Moreover, in some cases, other immunosuppressive medications were substituted with adalimumab due to inadequate clinical response [11]. Accordingly, MMF was substituted with adalimumab showing a remarkable clinical response with milder relapses after the steroid was tapered. During relapses, intralesional triamcinolone was injected into the ulcer periphery circumferentially, as it is effective in limited disease [12].

Colchicine is proposed to be a treatment option as an antineutrophilic agent in PG [2]. In the literature, only two case reports were treated initially with systemic steroids and then stabilized on colchicine alone, which was well-tolerated except for one patient who reported mild diarrhea. Furthermore, colchicine has a lower adverse effect on long-term use than MMF [13,14]. For our patients, we use colchicine in combination with adalimumab as it works effectively in stabilizing refractory PG.

## Conclusions

In conclusion, the choice of therapy is individual to each patient and is based on their response as well as their association with other systemic diseases. To add to the current literature, we proposed that both colchicine and adalimumab could be excellent adjunctive therapeutic options besides corticosteroids in the treatment of refractory PG. Additional studies on a larger scale are required to determine the most effective course of treatment for PG.

## References

[1] Bolognia J, Schaffer J, Cerroni J (2018). Dermatology. https://www.elsevier.com/books/dermatologia/bolognia/978-84-9113-365-0.

[2] Maronese CA, Pimentel MA, Li MM, Genovese G, Ortega-Loayza AG, Marzano AV (2022). Pyoderma gangrenosum: an updated literature review on established and emerging pharmacological treatments. Am J Clin Dermatol.

[3] Rozen SM, Nahabedian MY, Manson PN (2001). Management strategies for pyoderma gangrenosum: case studies and review of literature. Ann Plast Surg.

[4] Marzano AV, Trevisan V, Lazzari R, Crosti C (2011). Pyoderma gangrenosum: study of 21 patients and proposal of a 'clinicotherapeutic' classification. J Dermatolog Treat.

[5] Maverakis E, Ma C, Shinkai K (2018). Diagnostic criteria of ulcerative pyoderma gangrenosum: a Delphi consensus of international experts. JAMA Dermatol.

[6] Adachi Y, Kindzelskii AL, Cookingham G, Shaya S, Moore EC, Todd RF 3rd, Petty HR (1998). Aberrant neutrophil trafficking and metabolic oscillations in severe pyoderma gangrenosum. J Invest Dermatol.

[7] Ahronowitz I, Harp J, Shinkai K (2012). Etiology and management of pyoderma gangrenosum: a comprehensive review. Am J Clin Dermatol.

[8] Ashchyan HJ, Butler DC, Nelson CA (2018). The association of age with clinical presentation and comorbidities of pyoderma gangrenosum. JAMA Dermatol.

[9] Li J, Kelly R (2013). Treatment of pyoderma gangrenosum with mycophenolate mofetil as a steroid-sparing agent. J Am Acad Dermatol.

[10] Hrin ML, Bashyam AM, Huang WW, Feldman SR (2021). Mycophenolate mofetil as adjunctive therapy to corticosteroids for the treatment of pyoderma gangrenosum: a case series and literature review. Int J Dermatol.

[11] McKenzie F, Cash D, Gupta A, Cummings LW, Ortega-Loayza AG (2019). Biologic and small-molecule medications in the management of pyoderma gangrenosum. J Dermatolog Treat.

[12] Prajapati V, Man J, Brassard A (2009). Pyoderma gangrenosum: common pitfalls in management and a stepwise, evidence-based, therapeutic approach. J Cutan Med Surg.

[13] Kontochristopoulos GJ, Stavropoulos PG, Gregoriou S, Zakopoulou N (2004). Treatment of Pyoderma gangrenosum with low-dose colchicine. Dermatology.

[14] Paolini O, Hebuterne X, Flory P, Charles F, Rampal P (1995). Treatment of pyoderma gangrenosum with colchicine. Lancet.

